# IFN-Gamma-Dependent and Independent Mechanisms of CD4^+^ Memory T Cell-Mediated Protection from *Listeria* Infection

**DOI:** 10.3390/pathogens7010022

**Published:** 2018-02-13

**Authors:** Stephanie M. Meek, Matthew A. Williams

**Affiliations:** Department of Pathology, University of Utah, Salt Lake City, UT 84112, USA; stephanie.meek@path.utah.edu

**Keywords:** immunological memory, secondary challenge, CD4^+^ T cells, *Listeria monocytogenes*

## Abstract

While CD8^+^ memory T cells can promote long-lived protection from secondary exposure to intracellular pathogens, less is known regarding the direct protective mechanisms of CD4^+^ T cells. We utilized a prime/boost model in which mice are initially exposed to an acutely infecting strain of lymphocytic choriomeningitis virus (LCMV), followed by a heterologous rechallenge with *Listeria monocytogenes* recombinantly expressing the MHC Class II-restricted LCMV epitope, GP_61–80_ (Lm-gp61). We found that heterologous Lm-gp61 rechallenge resulted in robust activation of CD4^+^ memory T cells and that they were required for rapid bacterial clearance. We further assessed the relative roles of TNF and IFNγ in the direct anti-bacterial function of CD4^+^ memory T cells. We found that disruption of TNF resulted in a complete loss of protection mediated by CD4^+^ memory T cells, whereas disruption of IFNγ signaling to macrophages results in only a partial loss of protection. The protective effect mediated by CD4^+^ T cells corresponded to the rapid accumulation of pro-inflammatory macrophages in the spleen and an altered inflammatory environment in vivo. Overall, we conclude that protection mediated by CD4^+^ memory T cells from heterologous *Listeria* challenge is most directly dependent on TNF, whereas IFNγ only plays a minor role.

## 1. Introduction

A hallmark of adaptive immunity is the formation of memory following immunization or infection. Memory T cells, once formed, survive stably in both mice and humans and are a key component of protective immunity, responding more rapidly and robustly to secondary challenge [1,2,3,4,5]. A large number of studies have assessed the effector functions by which CD8^+^ memory T cells mediate protection from secondary exposure to viral or cytosolic bacterial pathogens, but studies of the role of CD4^+^ memory T cells have more closely focused on their helper role in enhancing CTL and antibody responses. In order to directly analyze the properties and functions of secondary CD4^+^ T cell responses, we utilized a prime/boost model in which mice are initially exposed to an acutely infecting strain of lymphocytic choriomeningitis virus (LCMV), followed by a heterologous rechallenge 6–8 weeks later with *Listeria monocytogenes* recombinantly expressing the immunodominant I-A^b^-restricted LCMV epitope, GP_61–80_ (Lm-gp61). Heterologous Lm-gp61 rechallenge resulted in robust secondary expansion of GP_61–80_-specific CD4^+^ memory T cells, enhanced secondary effector function as compared to homologous rechallenge with LCMV, and stable long-lived persistence of secondary CD4^+^ memory T cells [2,6]. Furthermore, we found that CD4^+^ memory T cells were capable of providing direct protection from a heterologous Lm-gp61 rechallenge [7]. Because protection in this model system was independent of CD8^+^ T cells and antibodies, in the current study we have employed it to test mechanisms of direct protection mediated by CD4^+^ memory T cells against the intracellular bacterium *Listeria monocytogenes* (Lm).

Several properties highlight the enhanced effector functions of CD4^+^ memory T cells. First, CD4^+^ memory T cells are highly sensitive to low antigen concentrations, allowing them to respond rapidly upon secondary infection [5,7,8]. Second, CD4^+^ memory T cell are able to immediately produce multiple cytokines, including IFNγ, IL-2 and TNF, leading to a profound alteration of the early inflammatory environment following infection [7], and the presence of CD4^+^ memory T cells with the ability to produce multiple cytokines is correlated to enhanced protection from secondary challenge [9,10]. Third, CD4^+^ memory T cells can home to tissue sites of infection. For example, CD4^+^ memory T cells can be detected in the liver many months after Lm infection and tissue-homing and/or tissue-resident CD4^+^ memory T cells have been characterized for a variety of infections in the lung, skin and reproductive tract [11,12,13,14,15,16]. Lastly, CD4^+^ memory T cells can rapidly re-express effector molecules such as Granzyme B and exert cytolytic function [17,18,19], although these findings are mostly limited to anti-viral responses.

In a prior study we found that the secondary effector function of CD4^+^ memory T cells was highly dependent on the inflammatory environment, as disruption of IL-12 and Type I IFN had opposing effects on effector differentiation of CD4^+^ memory T cells [7]. Further exploration of how the inflammatory environment influences secondary responses by CD4^+^ T cells will allow for a better understanding of how current vaccine strategies, particularly booster vaccinations which result in secondary responses and secondary memory formation, impact CD4^+^ memory T cell protective function.

While CD4^+^ T cells are required for optimal generation and maintenance of CD8^+^ memory T cells [20,21,22,23], protection from primary and secondary Lm infection mediated by CD8^+^ T cells plays a more dominant role than CD4^+^ T cells. CD8^+^ T cells can mediate primary and protective immunity in a manner that is independent of Perforin and IFNγ but dependent on TNF [24,25,26], although other studies have indicated a potential role for both Perforin and IFNγ in CD8-mediated immunity [27,28]. The key role of TNF is further reinforced by the finding that patients receiving TNF inhibitor treatment for chronic inflammatory conditions are more susceptible to infection with intracellular bacteria, including Lm [29,30]. In contrast, CD4^+^ T cell-mediated protection was reported to require IFNγ [31]. However, these studies depended on adoptive transfer models that may not have replicated the in vivo inflammatory environment induced by endogenously arising CD4^+^ T cell responses. Both IFNγ and TNF are key components of the innate response, with genetic disruption of these pathways resulting in early lethality following Lm infection [26,32,33]. IFNγ is a well-studied inducer of macrophage activation, and Type I IFN, a known antagonist of protective immunity to Lm [34,35], acts in part by inducing down-regulation of the IFNγR on macrophages [36]. Clearly, both TNF and IFNγ play key roles in the adaptive and innate arms of the protective immune response to Lm. However, it remains unclear whether endogenously arising CD4^+^ memory T cells mediate protective immunity via IFNγ, as during the primary response, or whether they adopt distinct mechanisms of protection.

Due to our observation that CD4^+^ memory T cells are sufficient to mediate rapid clearance of secondary Lm infection, we sought to define the mechanisms behind their protective effect. In a variety of model systems, the mechanism underlying the protective function of both innate and adaptive immune cells, including CD4^+^ T cells, are distinct during the primary versus secondary immune response [37,38,39]. We utilized a heterologous rechallenge model wherein mice are initially infected with LCMV and allowed at least 30 days for pathogen clearance and memory formation. Subsequently, mice were rechallenged with Lm-gp61, allowing for the specific induction of recall responses by I-A^b^/GP_61–80_-specific CD4^+^ memory T cells without substantial contribution from the CD8^+^ memory T cell compartment. Because previous studies with Lm have highlighted the critical roles of IFNγ and TNF in protective CD4^+^ or CD8^+^ T cell function during primary responses, we hypothesized that CD4^+^ memory T cells would mediate protection following heterologous rechallenge primarily via the production of IFNγ. Conversely, TNF is required for secondary protection mediated by CD8^+^ T cells [24,25]. Additionally, SCID mice showed impaired clearance at day 3 post-infection when treated with TNF neutralizing antibodies, and by day 5 most anti-TNF treated animals had succumbed to infection [40], showing a requirement for TNF in both the innate and adaptive arms of the immune response. Therefore, we also considered the possibility that CD4^+^ memory T cells could mediate enhanced Lm clearance via early production of TNF. 

We found that CD4^+^ memory T cell-dependent protection from heterologous challenge with Lm-gp61 was only somewhat dependent on the presence of IFNγ. Even macrophage-specific deletion of IFNγR resulted in only a modest loss of protection following secondary challenge. Conversely, neutralization of TNF resulted in a severe reduction in protection following heterologous rechallenge. Accumulation and activation of IFNγR-expressing M1 phenotype macrophages during the secondary response required the presence of CD4^+^ memory T cells and TNF. Overall these results point to a substantially greater role for TNF than IFNγ in CD4^+^ memory T cell-mediated protection from heterologous Lm-gp61 rechallenge and suggest that the mechanisms of protection mediated by CD4^+^ T cells during primary and secondary challenges differ.

## 2. Results

### 2.1. CD4^+^ Memory T Cells Induce Rapid Clearance of a Heterologous Lm-gp61 Challenge

**Figure 1:**
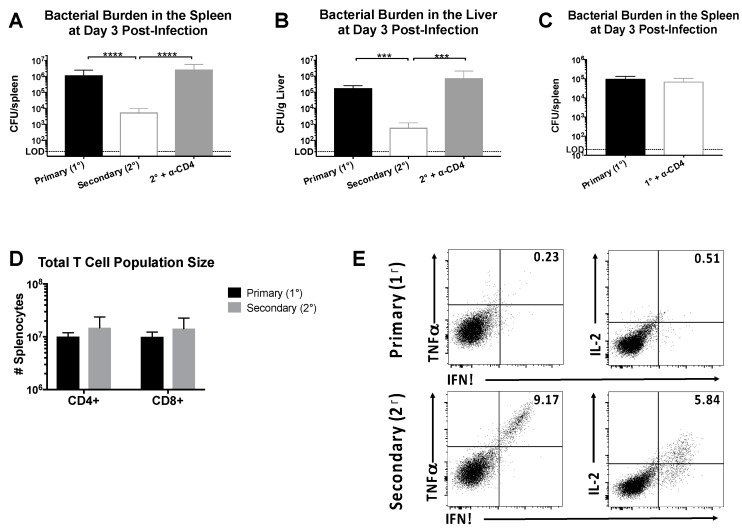
CD4^+^ memory T cells induce rapid clearance following Lm-gp61 rechallenge. Naïve or lymphocytic choriomeningitis virus (LCMV)-immune (>day 30 post-infection) B6 mice were infected with Lm-gp61. Additionally, some LCMV-immune mice were treated with anti-CD4 antibody to deplete CD4^+^ T cells prior to challenge. Bacterial burden in the (**A**) spleen (CFU/spleen) and (**B**) liver (CFU/g) was measured 3 days post-challenge; (**C**) Bacterial burden in the spleen of naïve mice with and without CD4 depletion, infected with Lm-gp61 was measured 3 days post-challenge; (**D**) Total numbers of CD4^+^ and CD8^+^ T cells in the spleen at day 3 post-challenge of naïve and LCMV-immune mice; (**E**) At day 3 post-challenge, splenocytes were stimulated *ex vivo* with the LCMV peptide GP_61–80_ in the presence of Brefeldin A, then fixed and permeabilized and stained with antibodies to detect the presence of intracellular cytokines. Representative flow plots are gated on total CD4^+^ T cells. *n* = 4–5 mice/group, data are representative of two separate experiments.

We sought to define the protective capacity of CD4^+^ memory T cells in LCMV-immune mice receiving a heterologous rechallenge with Lm-gp61. In our previous studies, we performed adoptive transfer experiments to demonstrate a direct role for CD4^+^ memory T cells in mediating protection. However, protection in our heterologous prime/boost model (LCMV → Lm-gp61) could be complicated by the presence of a CTL response to a previously described minor MHC Class I-restricted epitope within GP_61–80_ [41]. To address this issue, B6 mice were infected with LCMV, then challenged with Lm-gp61 >30 days later following treatment with anti-CD4-depleting or PBS control. By day 3 post-challenge with Lm-gp61, LCMV-immune mice cleared infection more rapidly in both the spleen and liver (Figure 1A,B). Addition of an isotype control during either primary infection or heterologous rechallenge had no effect on clearance (data not shown). The protective effect was dependent upon the presence of CD4^+^ T cells, as mice treated with CD4-depleting antibody prior to infection had bacterial loads similar to a primary infection (Figure 1A,B). This effect is specifically due to the presence of memory CD4^+^ T cells in immune mice and not myeloid cells expressing CD4, as depletion of CD4^+^ T cells in a naïve host had no effect on pathogen load and bacterial clearance (Figure 1C). Depletion of CD8^+^ T cells prior to rechallenge resulted in no significant difference in protection (data not shown), confirming the dominant role for CD4^+^ memory cells in protection from heterologous rechallenge with Lm-gp61. There was no change in the total number of CD4^+^ or CD8^+^ T cells when comparing primary versus secondary challenge (Figure 1D) but rather an increase in the number of antigen-specific secondary effector cells. In untreated LCMV-immune mice large numbers of secondary effector CD4^+^ T cells were present in the spleen and able to produce high levels of multiple cytokines upon restimulation, including IFNγ, TNFα and IL-2 (Figure 1E). These results confirm that rapid clearance following heterologous rechallenge is dependent on the presence of CD4^+^ memory T cells.

### 2.2. Heterologous Secondary Challenge with Lm-gp61 Induces an Altered Inflammatory Environment Compared to Primary Lm-gp61 Infection

**Figure 2:**
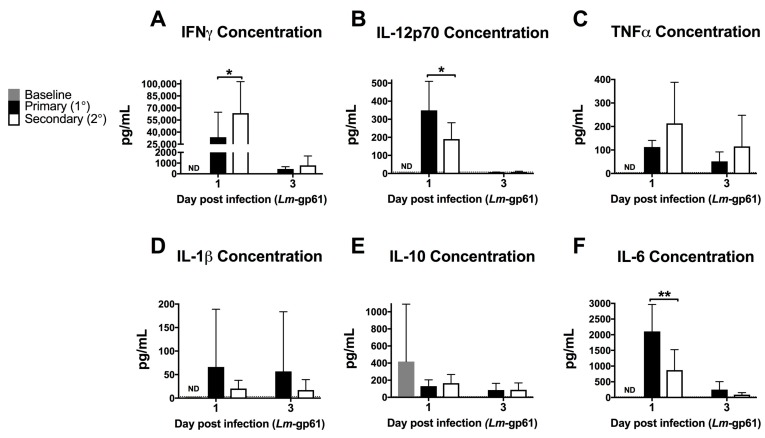
Heterologous challenge with Lm-gp61 induces increased levels of serum IFNγ. Naïve and LCMV-immune mice were challenged with Lm-gp61. Cytokine concentrations in the serum at day 1 and 3 post-infection were assessed using a cytokine bead array for (**A**) IFNγ; (**B**) IL-12p70; (**C**) TNF; (**D**) IL-1β; (**E**) IL-10; and (**F**) IL-6. *n* = 8 mice/group, data are pooled from two separate experiments. Dotted lines indicate limit of detection for the assay.

To assess the impact of the rapid response of CD4^+^ memory T cells on the early inflammatory environment following rechallenge, we measured the levels of inflammatory cytokines in the serum after primary Lm-gp61 challenge or secondary heterologous Lm-gp61 challenge. Heterologous rechallenge of CD4^+^ memory T cells responses induced significantly higher levels of IFNγ. TNF in the serum was not significantly different by 24 h post-infection, as compared to primary challenge with Lm-gp61. This may reflect differences in local versus systemic concentrations of TNF. In contrast, heterologous challenge resulted in reduced induction of systemic IL-12 and IL-6, with no significant differences in the levels of IL-1 and IL-10 (Figure 2A–F). It has long been appreciated that IFNγ is a critical cytokine in mediating protection from primary Lm infection [26] and higher serum IFNγ levels correlate to more rapid clearance in our model. In contrast, levels of IL-12p70 are significantly lower in a secondary infection, indicating that CD4^+^ memory T cell-dependent induction of elevated IFNγ is likely IL-12-independent (Figure 2B). Despite the previously described central role of TNF in protection from Lm, differences in systemic TNF levels between primary and secondary infections were not significant (Figure 2C). Although IL-6 is another key cytokine required for protection from primary Lm infection [42,43], we observed lower levels of systemic IL-6 following heterologous Lm-gp61 challenge, as compared to primary infection (Figure 2C). Overall, these differences highlight the unique inflammatory environment induced by the secondary response of CD4^+^ memory T cells, suggesting the possibility that inflammatory cytokines could have unique roles in these settings.

### 2.3. Rapid Clearance Following Heterologous Lm-gp61 Challenge Is Highly Dependent on TNF But Only Partly Dependent on IFNγ Signaling to Myeloid Cells

**Figure 3:**
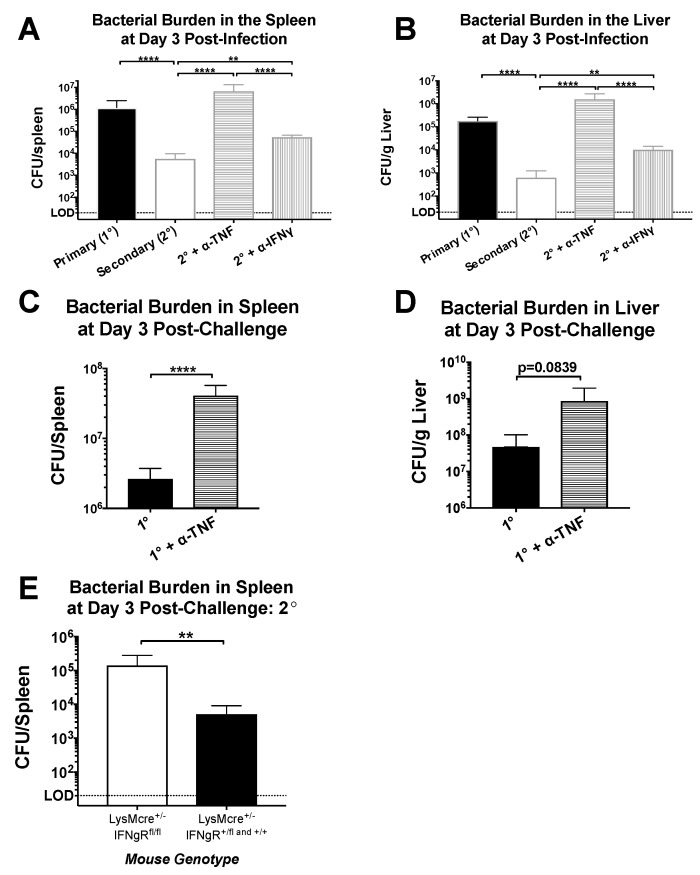
Protection mediated by CD4^+^ memory T cells is heavily dependent on TNF but only partly dependent on IFNγ. Naïve and LCMV-immune mice were challenged with Lm-gp61 and some groups of mice were additionally treated with neutralizing antibodies to IFNγ or TNF. Bar graphs indicate bacterial load in the (**A**) spleen and (**B**) liver day 3 following primary or secondary challenge (*n* = 4–7 mice/group); (**C**,**D**) Bar graphs indicate bacterial load in the spleen and liver, as indicated, on day 3 following primary Lm-gp61 infection with or without TNF neutralization (*n* = 4 mice/group); (**E**) Bar graph shows bacterial burden in the spleen of LCMV-immune *LysM^Cre^/Ifngr1^fl/fl^* mice day 3 after rechallenge with Lm-gp61 (*n* = 4 mice/group). Data are representative of at least two separate experiments.

As noted previously, both IFNγ and TNF are produced at high levels by CD4^+^ memory T cells after heterologous challenge. To test the role of these cytokines in this setting, we challenged LCMV-immune mice with Lm-gp61 as before, with some groups receiving TNF- or IFNγ-neutralizing antibody treatments, or PBS control. While IFNγ neutralization resulted in a somewhat higher bacterial load by day 3 post-infection, the effect was modest and not statistically significant. In contrast, TNF neutralization resulted in a complete loss of protection (Figure 3A,B). Neutralization of TNF or IFNγ during a primary Lm-gp61 infection resulted in dramatically increased susceptibility to infection in LCMV-naïve mice, indicating that neutralization of either cytokine was sufficient to reduce protection from a primary response (Figure 3C,D, data not shown). Addition of isotype controls did not impact clearance (data not shown). Bacterial loads in untreated animals were consistent with our previously published results [7]. Given the complex immunomodulatory roles of IFNγ in vivo, we further tested whether IFNγ signaling directly to macrophages played a key role in protection. We crossed mice containing LoxP sites flanking the IFNγR1 locus with mice expressing Cre under the control of the LysM promoter in order to generate a scenario in which only IFNγ signaling to myeloid cells is disrupted. We then infected these mice with LCMV, which clear primary LCMV infection normally [44]. Upon heterologous challenge of these mice with Lm-gp61 30 days later, we observed a partial loss of protection in mice lacking IFNγR1 expression on myeloid lineage cells. Overall, we concluded that TNF plays a dominant role in CD4^+^ memory T cell-mediated protection from secondary challenge, whereas IFNγ signaling to macrophages (and other myeloid cells) plays a significant but less dominant role. The TNF-dependent role in the protective effect mediated by CD4^+^ memory T cells is a function previously attributed to CD8^+^ T cells. These results also highlight key differences in the requirement for IFNγR1 expression by macrophages during primary versus secondary Lm infection [45].

### 2.4. TNF But Not IFNγ Is Required for Macrophage Activation Early in Secondary Infection

**Figure 4:**
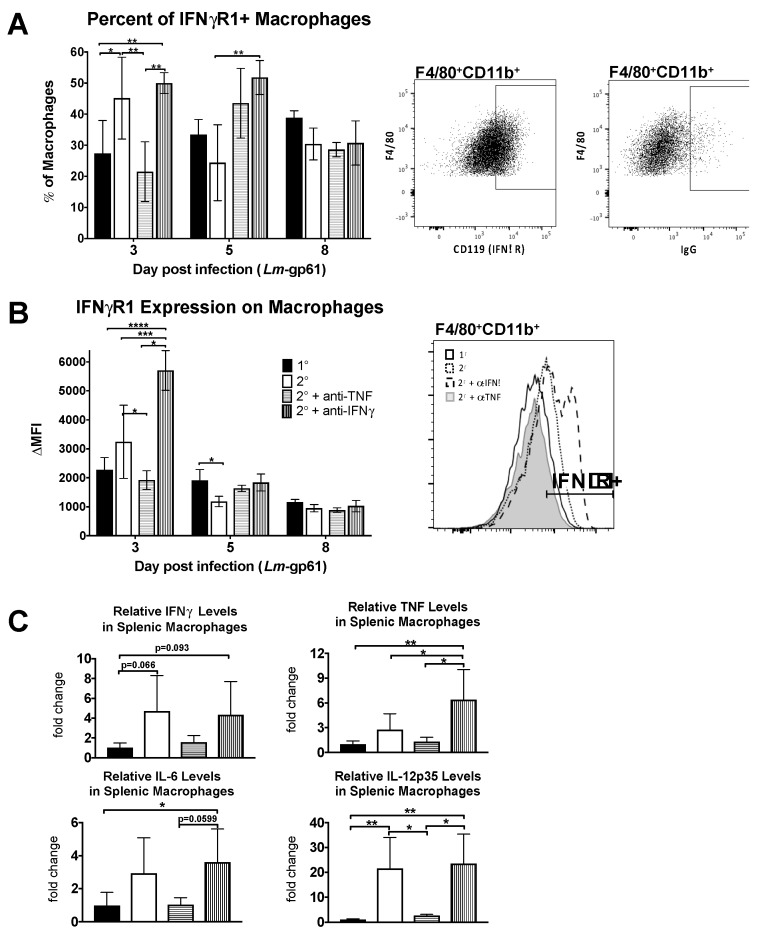
Neutralization of TNF prevents accumulation of activated macrophages in the spleen. Naïve and LCMV-immune mice were challenged with Lm-gp61 and some groups of mice were additionally treated with neutralizing antibodies to IFNγ or TNF. (**A**) Splenocytes were gated for F4/80^+^CD11b^+^ macrophages. The bar graph indicates the frequency of macrophages expressing IFNγR1 (CD119) for each treatment group. Accompanying flow plots indicate representative gating for CD119^+^ macrophages, as compared to isotype control. Frequencies were obtained by subtracting the percentage that were stained by the isotype control; (**B**) The bar graph indicates the change in MFI of CD119 staining on F4/80^+^CD11b^+^ macrophages for each treatment group. The change in MFI was obtained by subtracting the MFI following isotype control staining from the MFI following CD119 staining. The accompanying flow plot indicates representative staining for CD119 on macrophages for each treatment group; (**C**) RNA from FACS-sorted F4/80^+^CD11b^+^ macrophages was analyzed by semi-quantitative RT-PCR for changes in cytokine transcript levels. Bar plots indicate the relative fold change in expression between treatment groups for the indicated transcripts. *n* = 3–5 mice per group.

To determine what impact TNF signaling has on macrophage activation in vivo, we examined the accumulation of IFNγR-expressing macrophages in the spleen as well as the upregulation of IFNγR1 on their cell surface. We observed both a decrease in the frequency of IFNγR1-expressing macrophages in the spleen as well as a decrease in the intensity of IFNγR1 cell surface staining on day 3 following TNF neutralization during heterologous Lm-gp61 challenge (Figure 4A,B). In contrast IFNγ neutralization resulted in no change in the percent of IFNγR1^+^ macrophages, though the surface expression increased on a per cell basis. This may be a result of cytokine neutralization, as binding of IFNγ to its receptor results in receptor internalization and lower surface expression [46]. Differences in the accumulation of IFNγR-expressing macrophages occurs only early in infection, as by 8 days post-infection there are no significant differences between treatment groups (Figure 4A,B).

To examine functional changes in macrophages, we sorted F4/80^+^CD11b^+^ cells from the spleens of LCMV-immune mice 3 days after heterologous challenge with Lm-gp61 and isolated RNA for semi-quantitative RT-PCR analysis. While we did not observe significant differences in expression of IFNγ, TNF or IL-6, as compared to macrophages isolated after primary Lm-gp61 infection, heterologous challenge resulted in a significant induction in IL-12p35 expression by splenic macrophages. This induction was completely abrogated following TNF neutralization but unaffected by IFNγ neutralization (Figure 4C). These findings further support our conclusion that TNF neutralization results in impaired accumulation and activation of macrophages following heterologous Lm-gp61 challenge.

### 2.5. IFNγ-Dependent Regulation of IL-6 Does Not Impact Protection from Heterologous Lm-gp61 Challenge

**Figure 5:**
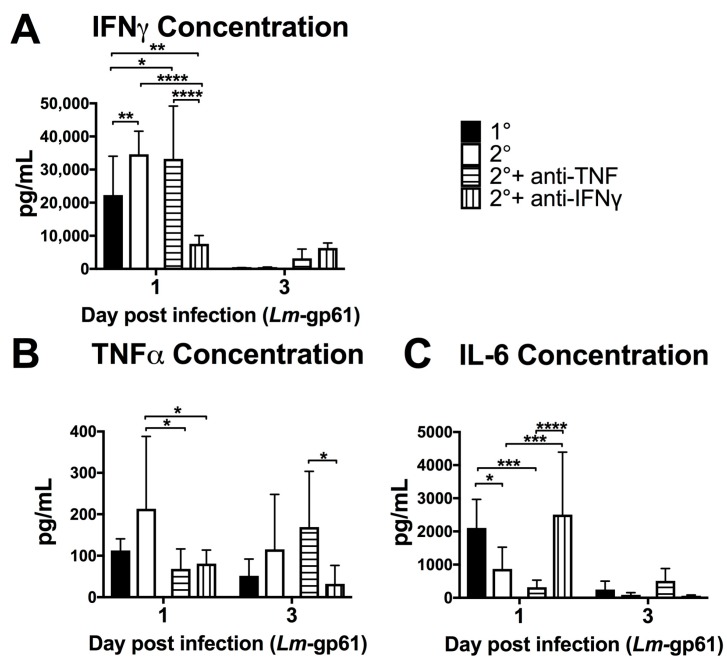
Neutralization of IFNγ and TNF alter the inflammatory environment induced by secondary challenge. Naïve and LCMV-immune mice were challenged with Lm-gp61 and some groups of mice were additionally treated with neutralizing antibodies to IFNγ or TNF. Bar plots show the concentration of (**A**) IFNγ; (**B**) TNF and (**C**) IL-6 in the serum of the indicated treatment groups at day 1 or 3 after Lm-gp61 challenge. *n* = 7–8 mice per group, data are representative of two separate experiments.

In order to better understand the impact of TNF and IFNγ on systemic inflammatory responses, we measured the concentrations of serum cytokines on days 1 and 3 following heterologous rechallenge in the presence of TNF or IFNγ neutralizing antibodies (Figure 3). Neutralization of TNF resulted in no change to systemic IFNγ levels (Figure 5A) at day 1 post-challenge. Conversely, IFNγ neutralization resulted in a significant decrease in circulating TNF levels (Figure 5B). This again reflects differences in the systemic and local inflammatory environments, as the serum concentration of TNF does not reflect the increase in TNF production by splenic macrophages (Figure 4C). TNF and IFNγ had opposite effects on IL-6 production, with IFNγ neutralization resulting in a significant increase in IL-6, with concentrations similar to that of a primary Lm-gp61 infection. We additionally neutralized IL-6 during heterologous rechallenge, with no effect on bacterial clearance (data not shown). Previous work had indicated an important protective role for IL-6 during in primary infection [42,43] and these results suggest that whereas IFNγ may be an important regulator of IL-6 during primary infection, its role is diminished due to the effector function of secondary CD4^+^ effector T cells induced by heterologous rechallenge.

## 3. Discussion

We demonstrate that CD4^+^ memory T cells alone are sufficient to protect from a Lm challenge in a manner independent of both CTLs and antibodies. Additionally, we have provided evidence that both TNF and IFNγ contribute to this protective effect, with TNF playing a dominant role and IFNγ playing a minor role. While much of the IFNγ is contributed by the innate immune response, IFNγ-dependent mechanisms are a key component of the contribution of Th1 cells to control of primary Lm infection [31]. We observe a quite different role for IFNγ in protection mediated by CD4^+^ memory T cells following heterologous rechallenge. CD4^+^ memory T cells mediate protection in a largely IFNγ-independent fashion and the presence of CD4^+^ memory T cells is sufficient to render IFNγ dispensable for Lm clearance. Our data suggest that the contribution of CD4^+^ memory T cells to protection from secondary challenge may be more similar to that of CD8^+^ T cells, with TNF playing a dominant role. These findings particularly highlight that the mechanisms of protection mediated by T cells during primary and secondary responses can differ, illustrating the need to better understand the functional mechanisms underlying memory T cell-mediated bacterial clearance in the development of effective vaccination strategies. Based on our results, we propose a model in which protection mediated by CD4^+^ T cells during primary Lm infection is primarily IFNγ-dependent, whereas protection mediated by CD4^+^ memory T cells following heterologous rechallenge is primarily TNF-dependent. It will be critical in future studies to determine whether similar mechanisms of protection are employed by CD4^+^ memory T cells during the antiviral response.

As evidenced here, TNF is of particular importance in CD4^+^ memory T cell-mediated protection, as the absence of TNF results in a loss of protection during heterologous secondary challenge. Studies involving primary Lm responses describe a role for TNF in primary protection, as TNF neutralization or infection of gene knock-out mice result in increased bacterial burden and increased mortality [24,25]. Our data agree with this, as neutralization of TNF during primary Lm-gp61 infection resulted in increased bacterial burden 3 days post-challenge. While we observe a loss of protection at day 3 post-infection during secondary challenge in the absence of TNF, the levels of bacteria are similar to those of a normal primary infection, indicating an altered mechanism of action during a secondary infection compared to a primary infection. Additionally, TNF-dependent mechanisms of protection appear localized to the site of infection, as we observe no significant systemic changes in TNF during heterologous rechallenge but do observe induction in splenic macrophages and memory T cells. TNF neutralization results in impairment of macrophage activation, as evidenced by decreased expression of IL-12 and IFNγR1, a marker for classically activated pro-inflammatory macrophages. Like IFNγ, TNF plays key roles in both the adaptive and innate facets of the anti-Lm immune response. While TNF may play a role in regulating the IFNγ-dependent response, the relative role of IFNγ in mediating protection is minor. Future studies are required to determine whether it is the contribution of CD4^+^ memory T cells to the TNF response, the action of TNF on CD4^+^ memory T cells, or some other mechanism that promotes faster bacterial clearance. Our findings suggest the likelihood of additional TNF-independent, CD4^+^ memory T cell-dependent mechanisms of protection from heterologous Lm challenge.

While TNF, IL-12 and IFNγ are classically associated with protective immunity to *Listeria*, other cytokines have also been shown to play a role. Of note, IL-6 has been demonstrated to promote protection from primary Lm infection, with IL-6 neutralization resulting in increased bacterial burden [42,43]. In contrast, it has also been shown that acquired immunity does not require IL-6 [47], a finding that we confirm in the heterologous rechallenge model. Other cytokines may also play an important role in CD4^+^ T cell-mediated protection, possibly independently of TNF. While IL-12, IL-6 and IFNγ are all inducible by TNF, IL-18 is a cytokine upstream of TNF that has been described as playing a role in protection from *Listeria* infection [48]. Additional investigation of these cytokine pathways will further elucidate the role of TNF-dependent and independent cytokine signaling in CD4^+^ T cell-mediated protection. Overall, our observations support the idea that secondary CD4^+^ T cell responses are sufficient to alter the anti-Lm immune response in key ways. First, they induce altered cytokine production systemically in the very earliest stages of the anti-Lm response. Second, they lead to a more rapid accumulation of activated macrophages in the spleen. Third, they are positioned to contribute to the inflammatory response at a time point that is normally dominated by innate immune cells. Understanding these and other differences between secondary and primary CD4^+^ T cell responses and the unique mechanisms by which CD4^+^ T cells mediate protection, will allow for fine-tuning of vaccines and immunotherapeutics in order to better manipulate CD4^+^ T cell activity in vivo. 

## 4. Materials and Methods

*Mice and Infections*. 6–8-week-old C57BL/6J (B6) mice were purchased from Jackson Laboratories (Bar Harbor, ME, USA). C57BL/6N-IFNγr1^tm1.1Rds^/J (IFNγR^fl^; stock number 025394) mice were purchased from Jackson Laboratories and bred to mice expressing Cre under the control of the LysM promoter [49]. LCMV-Armstrong and Lm-gp61 were stored and propagated as previously described [2]. For primary infections with LCMV-Arm, mice were injected intraperitoneally (i.p.) with 2 × 10^5^ plaque forming units (PFU). For infections with Lm-gp61, bacteria were first grown to log phase in BHI media as determined by the O.D. at 600 nm (0.3–0.7). Mice were then injected intravenously (i.v.) with 2 × 10^5^ colony forming units (CFU). Primary infections with LCMV were done between 6–9 weeks of age, while primary and secondary infections with Lm-gp61 were done 6 weeks later, when mice were 10–15 weeks old. All experiments using animals were performed under a protocol approved by the University of Utah IACUC (Protocol #15-09004, approved 23 September 2015).

*Neutralizing antibody treatments.* 200 μg anti-CD4 depleting antibodies (BioXCell, Clone GK1.5) were given i.p. on day-2 and -1 prior to infection with Lm-gp61. 0.5 mg anti-TNF neutralizing antibodies (BioXCell, XT3.11) or anti-IL-6 (BioXCell, MP5-20F3) were given i.p. 1 day prior to infection and then every other day after that until sacrifice. 1 mg neutralizing antibodies to IFNγ (BioXCell, XMG1.2) were given i.p. 1 day prior to infection and then every 4 days after that until sacrifice. Treatment efficacy was confirmed by flow cytometry or by the ability of neutralizing cytokines to impair clearance of primary Lm infection. PBS control was not different from IgG1 or IgG2b isotype controls given at equivalent amounts as neutralizing or depleting antibodies and we have utilized this method of control previously [7], so PBS injection served as a control for all antibody treatments.

*Serum Cytokine Analysis*. Mice were bled on days 1 and 3 post-infection. Blood was allowed to clot at room temperature then spun at max speed in a microcentrifuge for 20 min. Serum was collected and stored at −20 °C. Serum cytokine concentrations were measured using a custom 6-plex LEGENDplex bead-based cytokine assay (Biolegend, San Diego, CA, USA; IFNγ 740153, IL-1B 740157, IL-6 740159, IL-10 740158, IL-12 (p70) 740156, TNF-a 740154, Standard 740371, Detection antibodies 740165, buffer set B 740373). 

*Tissue and cell preparations.* Whole spleens and liver portions were collected in the tissue culture hood in 2 mL sterile PBS. Livers were weighed and all organs were dissociated using frosted microscope slides. For assessment of bacterial load, serial 1:10 dilutions were performed in sterile PBS and aliquots were plated on brain heart infusion agar (BHI) agar plates. Plates were incubated at 37 °C overnight. Colony counts were reported as CFU/spleen or CFU/g of liver. For all other cell preparations, dissociated tissues were places in single cell suspension in DMEM containing 10% fetal bovine serum (FBS), L-glutamine and Pen/Strep. For detection of intracellular cytokines, splenocytes were incubated with GP_61–80_ peptide (GLKGPDIYKGVYQFKSVEFD) for 4 h in the presence of Brefeldin A (Golgi Plug), followed by fixation and permeabilization, according to the manufacturer’s instructions (BD Biosciences).

*Flow Cytometry and Cell Sorting*. Cells were suspended in PBS + 1% FBS, then stained with fluorescent dye-conjugated antibodies (anti-CD4, anti-CD8, anti-IFNγ, anti-TNFα, anti-IL-2, anti-CD11b, anti-F4/80, anti-IFNγR) (Biolegend, San Diego, CA, USA) for 20–40 min. Samples were collected on a LSRFortessa flow cytometer (BD Biosciences, San Jose, CA, USA) and analyzed using FlowJo (TreeStar, Ashland, OR, USA). For cell sorting, splenocytes were stained with antibodies specific to CD4, CD8, CD11b and F4/80 for sorting by a BD FACSAria II (BD Bioscience) at the University of Utah Flow Cytometry Core Facility. Macrophages were sorted by gating on CD4^neg^CD8^neg^CD11b^+^F4/80^+^ cells and sorting directly into Qiazol (Qiagen, Germantown, MD, USA).

*RNA Isolation and RT-PCR*. RNA macrophages were isolated using the miRNeasy kit (Qiagen, Germantown, MD, USA). cDNA was generated using the SuperScript III First-Strand Synthesis System (Thermo Fisher Scientific, Waltham, MA, USA). PCR was performed with the SYBR Green Master Mix (Thermo Fisher Scientific) and primers specific for our genes of interest (DNA Synthesis Core, University of Utah) using an LC480 PCR LightCycler (Roche Diagnostics, Indianapolis, IN, USA). The following primer sequences were used: 1L12p53: F- TGCCTTGGTAGCATCTATGAGG, R- CGCAGAGTCTCGCCATTATGAT; TNF: F- ATGAGCACAGAAAGCATGA, R- AGTAGACAGAAGAGCGTGGT; IFNγ: F- TTCTTCAGCAACAGCAAGGC, R- TCAGCAGCGACTCCTTTTCC; IL6: F-CCTCTGGTCTTCTGGAGTACC; R- ACTCCTTCTGTGACTCCAGC [50].

*Statistical Analysis*. Statistical significance was determined using the student’s *t*-test for two groups and ANOVA for more than two groups using GraphPad Prism 7 software (Graphpad, La Jolla, CA, USA). Graphs depict mean ± SD, with a *p* value of less than 0.05 being considered significant. *p* values are indicated as follows: * *p* < 0.05, ** *p* < 0.01, *** *p* < 0.001, **** *p* < 0.0001.
